# Irinotecan-platinum combination therapy for previously untreated extensive-stage small cell lung cancer patients: a meta-analysis

**DOI:** 10.1186/s12885-018-4715-9

**Published:** 2018-08-10

**Authors:** Fei Xu, Xiaoli Ren, Yuan Chen, Qianxia Li, Ruichao Li, Yu Chen, Shu Xia

**Affiliations:** 1 Department of Oncology, Tongji Hospital, Tongji Medical College of Huazhong University of Science and Technology, Wuhan, People’s Republic of China; 2 Department of Oncology, Affiliated Hospital of Hebei University of Engineering, Handan, People’s Republic of China

**Keywords:** Small cell lung cancer, Extensive-staged, Irinotecan, Etoposide, Meta-analysis

## Abstract

**Background:**

There is still a debate regarding whether regimens combining irinotecan with platinum could replace regimens combining etoposide with platinum, as first-line chemotherapy for extensive-stage small cell lung cancer (ES-SCLC). We performed a meta-analysis to compare these regimens as first-line chemotherapy for ES-SCLC.

**Methods:**

A literature search for randomized controlled trials was performed using the Cochrane Library, PubMed, and Embase. The inverse variance method was used to estimate summary hazard ratios and their 95% confidence intervals for overall survival and progression free survival. Relative risk was used to estimate the overall response rate, disease control rate, 1-year survival, 2-year survival, and adverse event data.

**Result:**

Nine randomized controlled trials (2451 patients) were included. Regimens combining irinotecan and platinum improved overall survival, progression-free survival and overall response rate compared to combination etoposide and platinum regimens. Meanwhile, superior progression-free survival and overall response rate outcomes were observed in the Asian subgroup of patients. These patients receiving a combination irinotecan and platinum regimen experienced grade 3–4 diarrhea more frequently and experienced less hematologic toxic events than the non-Asian groups.

**Conclusions:**

Our data suggest that a combination irinotecan and platinum regimen can prolong overall survival, progression-free survival and overall response rate for patients with ES-SCLC as compared to a combination etoposide and platinum regimen. And the Asian patients could benefit from irinotecan combined with platinum easier.

## Background

Lung cancer, which represents 13% of newly diagnosed cancers worldwide, is the most common tumor type [1]. Small cell lung cancer (SCLC) accounts for approximately 15% of new cases of annually diagnosed lung cancer, and up to 25% of lung cancer deaths each year [2]. Approximately two-thirds of patients with SCLC are diagnosed with extensive-stage disease [3], which is defined as disease dissemination beyond the ipsilateral hemithorax including malignant pleural or pericardial effusion or hematogenous metastases [4]. Over the past 20 years, the standard therapy for most patients with extensive-staged small cell lung cancer (ES-SCLC) has been either carboplatin or cisplatin in combination with etoposide (EP) [5]. In 2002, the Japan Clinical Oncology Group (JCOG-9511) first acquired evidence for superior outcomes following therapy with irinotecan in combination with cisplatin (IP). Nevertheless, a subsequent and larger study failed to validate the observed difference survival benefit in JCOG-9511 between the IP and EP treatment arms. In 2010, in a meta-analysis, Jiang et al. [6] concluded that IP may have an advantage in overall response and OS as compared to EP in patients with ES-SCLC, but did not find superior results in progression-free survival (PFS); however, the authors did not include ethnicity in their analysis. Therefore, our meta-analysis was performed based on these prior studies to compare the efficacies and toxicities of IP and EP in patients with ES-SCLC, and these parameters were further analyzed in patient subpopulations.

## Methods

### Search strategy and study selection

The Cochrane Library, PubMed, and Embase electronic databases were used to perform an electronic search by combining following words: “small cell lung cancer” or “small cell lung carcinoma,” “irinotecan” or “CPT-11,” and “etoposide” or “VP-16”. To limit publication bias, the search was limited to “randomized controlled trial” and no language, publishing time limitation, or other restrictions were imposed. We also searched the Physician Data Query registry of ClinicalTrials.gov (http://clinicaltrials.gov) to identify ongoing studies.

### Inclusion and exclusion criteria

Two reviewers (Fei Xu and Xiaoli Ren) independently reviewed all studies that met the following selection criteria: (1) all patients recruited in the study who were diagnosed SCLC were previously untreated; (2) the study compared IP regimens with EP regimens; and (3) the study was a randomized controlled clinical trial. Trials were excluded if they did not meet the above inclusion criteria. Disagreements were resolved by discussion or by consulting with a third reviewer.

### Information extraction and assessment of methodological quality

Two reviewers (Fei Xu and Xiaoli Ren) independently extracted the following information from the included studies: first author’s name, year of publication, country, sex, average age, number of patients, chemotherapy regimens, stage of disease, primary endpoint, and second endpoint as well as hazard ratios (HRs) and respective confidence intervals for OS and PFS, complete response, partial response, overall response rate (ORR), disease control rate (DCR), 1-year survival rate, and 2-year survival rate. If HRs were not available, we extracted vital data through survival curves using Engauge Digitizer Version 4.1 software and then calculated HRs by the Tierney method [7]. Common adverse events of grade 3–4 toxicity such as anemia, leucopenia, neutropenia, thrombocytopenia, diarrhea, febrile neutropenia, infection, alopecia, fatigue and drug-related death were also extracted according to National Cancer Institute-Common Toxicity Criteria.

Methodological quality was assessed independently according to the following items: random sequence generation, allocation concealment, blinding of participants and personnel, blinding of outcome assessment, incomplete outcome data, selective reporting, and other bias. Each item was judged as “low,” “high,” or “unclear.” Disagreements were resolved by discussion or consulting with a third reviewer.

### Statistical analysis

Review manager 5.3 was used to analyze and generate data. Heterogeneity was identified using a chi-square test, and I^2^ (*P* < 0.1 and I^2^ > 50%) indicated significant heterogeneity. In the event that obvious heterogeneity was deemed valid, the random-effects model was used. Otherwise, the fixed-effects model was employed. The HR was used for PFS and OS. For dichotomous data, relative risk (RR) was used for ORR, DCR, 1-year survival, 2-year survival, and adverse event data. A *P* value < 0.05 was considered statistically significant. HR > 1 reflects more deaths or progression in the EP arm. RR > 1 reflects more events in the IP arm.

## Results

### Identification of studies and study quality

**Fig. 1:**
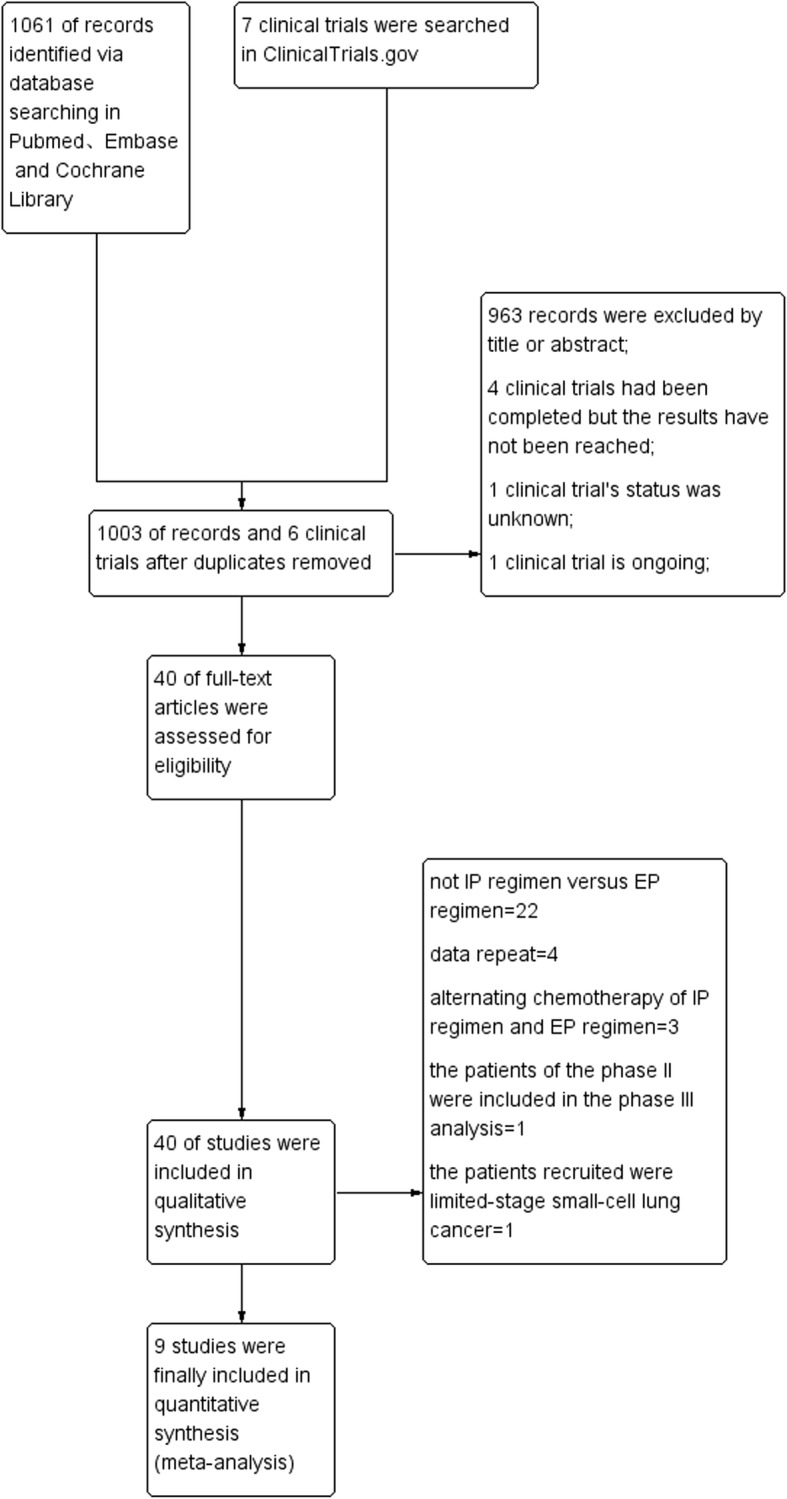
Flow chart showing the progression of trials through the review

**Fig. 2:**
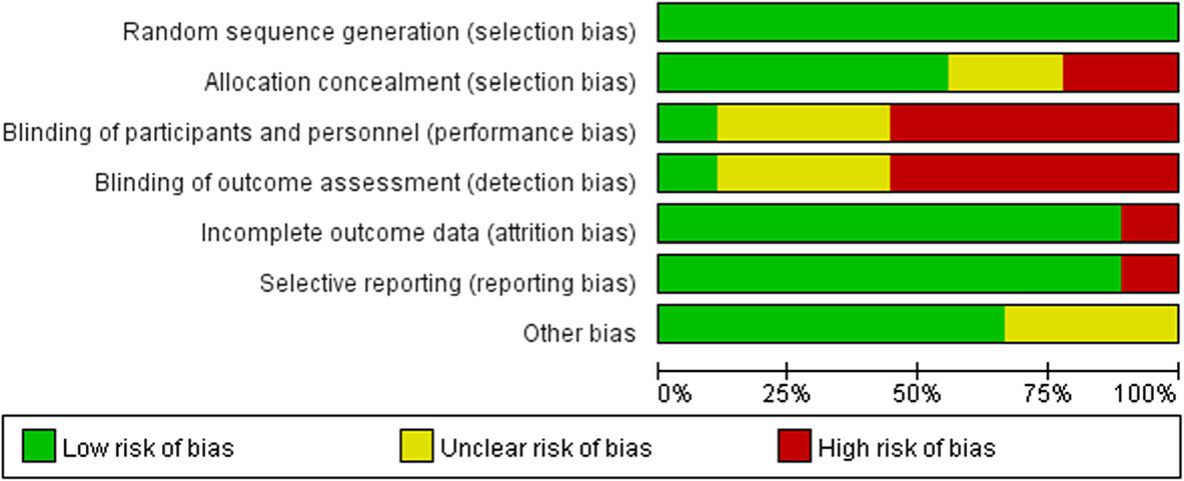
Risk of bias graph

**Fig. 3:**
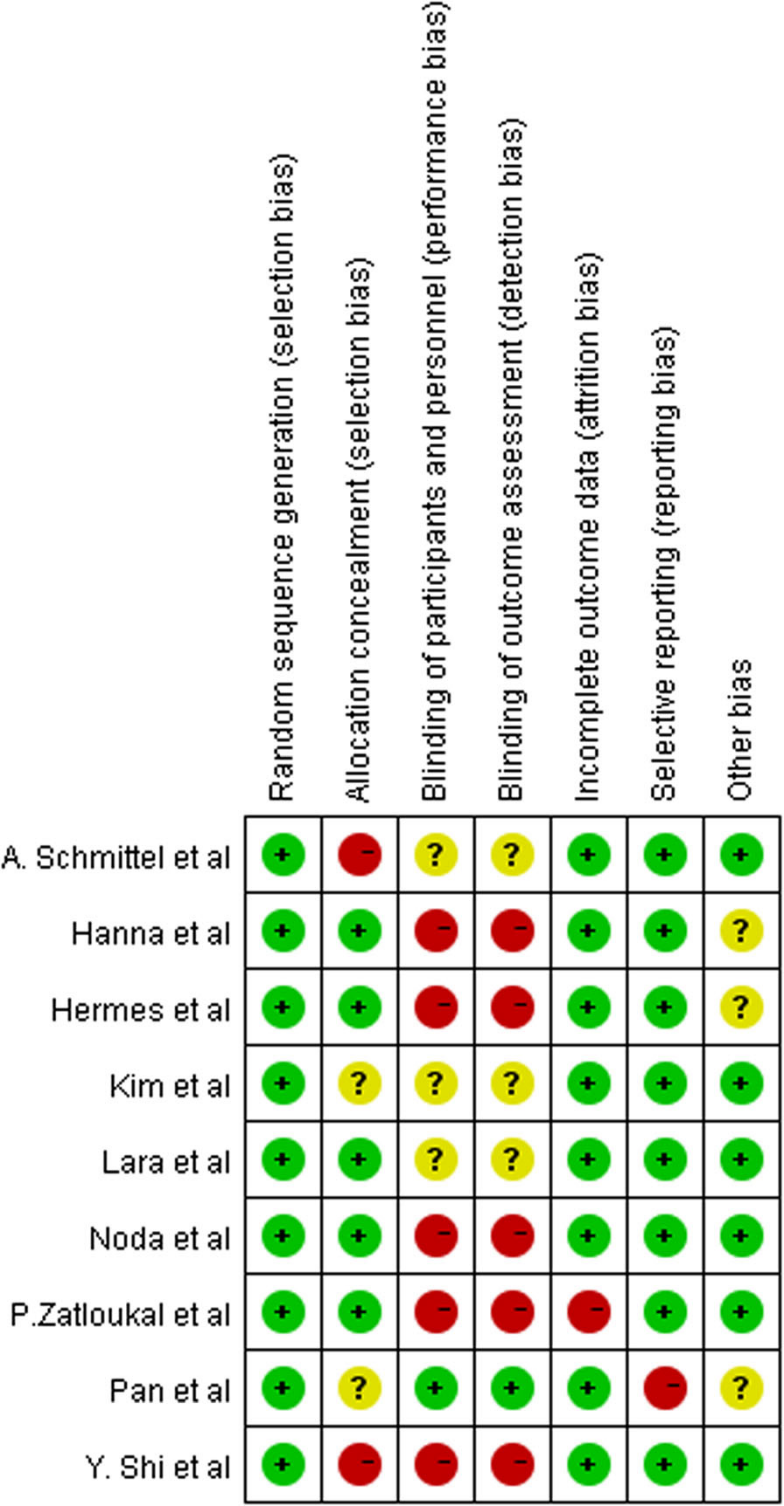
Risk of bias summary

We identified 1061 patient records, and seven clinical trials were identified on ClinicalTrials.gov according to the search strategy. After excluding duplicates, ongoing trials, trials of unknown status and results, and after screening titles and abstracts, 40 records were selected for full-text screening, of which nine publications [8–16] including 2451 patients that fulfilled all inclusion criteria were considered for analysis. A flow chart of our study is shown in Fig. 1. All identified studies were phase III randomized controlled trials. The included publications used cisplatin with two exceptions: Hermes et al. and Schmittel et al. used carboplatin. Detailed baseline characteristics of the included studies are presented in Table 1. According to the tool described in the Cochrane Handbook for Systematic Reviews of Interventions [17], we assessed the methodological quality of each included study (Figs. 2 and 3).

**Table 1:** Basic characteristics of the studies included in this meta-analysis

Author	Study ID	Country	Number	Regimens(number of arm)	Primary Endpoint	Second Endpoint	Gender M/F (IP VS EP)	Average Age (year)	PS (0–1)% (IP VS EP)
Kim et al	2018	Korea	362	IP: irinotecan 65 mg/m2 d1,8 cisplatin 70 mg/m2 d1(30) EP: etoposide 100 mg/m2 d1–3 cisplatin 70 mg/m2 d1(32)	OS	Toxicity、PFS、ORR、 CR、PR, etc	151/22 177/12	66 65	85.5 84.6
Y.Shi et al	2015	China	62	IP: irinotecan 65 mg/m2 d1,8 cisplatin 75 mg/m2 d1(30) EP: etoposide 100 mg/m2 d1–3 cisplatin 75 mg/m2 d1(32)	PFS	ORR、OS and toxicity	22/9 26/6	59 57	90 90.7
A.Schmittel et al	2011	German	216	IP: irinotecan 50 mg/m2 d1,8,15 carboplatin AUC 5 mg.min/ml(106) EP: etoposide 140 mg/m2 d1–3 carboplatin AUC 5 mg.min/ml(110)	PFS	OS、response rate and toxicity	70/36 71/39	60 63	80 80
P. Zatloukal et al	2010	predominantly European countries	405	IP: irinotecan 65 mg/m2 d1,8 cisplatin 80 mg/m2 d1(202) EP: etoposide 100 mg/m2 d1–3 cisplatin 80 mg/m2 d1(203)	OS	ORR、the duration of Response, etc	154/48 155/48	59 60	99 100
Lara et al	2009	American	651	IP: irinotecan 60 mg/m2 d1,8,15 cisplatin 60 mg/m2 d1(324) EP: etoposide 100 mg/m2 d1–3 cisplatin 80 mg/m2 d1(327)	Not State	Not State	188/136 182/145	62 63	100 100
Hermes et al	2008	Norway and Sweden	209	IC: irinotecan 175 mg/m2 d1 carboplatin AUC 4 mg.min/ml d1(105) EC: etoposide 120 mg/m2(orally) d1–5 carboplatin AUC 4 mg.min/ml d1(104)	OS	quality of life、CR	66/39 72/32	67 68	53 52
Pan et al	2006	China	61	IP: irinotecan 80 mg/ m2 d1,8,15 cisplatin 80 mg/ m2 d1–3(30) EP: etoposide 120 mg/ m2 d1–3 cisplatin 80 mg/ m2 d1–3(31)	Not State	Not State	24/6 23/8	54 51	100 100
Hanna et al	2006	American	331	IP: irinotecan 65 mg/m2 d1,8 cisplatin 30 mg/m2(221) EP: etoposide 120 mg/m2 d1–3 cisplatin 60 mg/m2(110)	OS	response rate、TTP	127/94 63/47	63 62	92.3 88.2
Noda et al	2002	Japan	154	IP: irinotecan 60 mg/m2 d1,8,15 cisplatin 60 mg/m2 d1(77) EP: etoposide 100 mg/m2 d1–3 cisplatin 80 mg/m2 d1(77)	OS	CR、ORR、PFS, etc	63/14 69/8	63 63	92 87

I irinotecan, P cisplatinum, E etoposide, C carboplatin, OS overall survival, PFS progression free survival, ORR overall response rate, CR complete remission, PR partial remission, TTP time to progession, PS performance status

### Overall survival

**Fig. 4:**
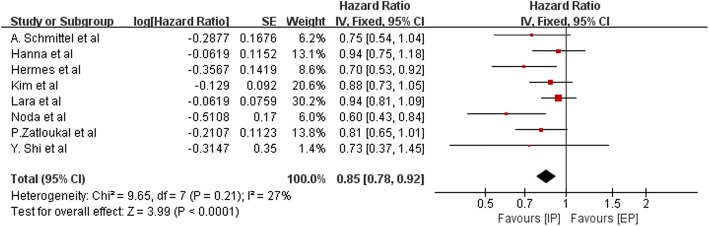
Forest plots estimating OS in IP vs EP

**Fig. 5:**
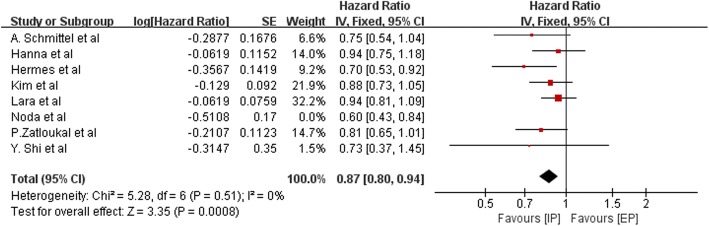
Sensitivity analysis of OS was employed to explore sources of heterogeneity. Heterogeneity declined from I^2^ = 27% to I^2^ = 0% when the study performed by Noda et al. was excluded

HRs for OS data were available for eight trials that altogether included 2390 patients (when data was acquired indirectly, HR was calculated by the Tierney method). The pooled HR was 0.85, indicating that an IP regimen likely prolongs OS in patients with SCLC (HR = 0.85; 95% CI, 0.78–0.92; P<0.0001; Fig. 4). The heterogeneity test (Chi^2^ = 9.65; *P* = 0.21; I^2^ = 27%) indicated that mild heterogeneity was present among the included studies; thus, the fixed-effects model was used. Although no significant heterogeneity was observed in this comparison, we performed subgroup analyses stratified by the use of platinum and patient ethnicity (Asian or non-Asian), and sensitivity analysis was employed to explore sources of heterogeneity. The details of subgroup analysis are listed in Table 2. We did not find obvious differences in heterogeneity, with one following exception: when the study performed by Noda et al. was excluded, heterogeneity declined from I^2^ = 27% to I^2^ = 0% (Fig. 5).

**Table 2:** The outcome of subgroup analysis stratified by platinum regimen and ethnicity

Subgroups	Pooled HR	95%CI	*P*-Value	I^2^ For Homogeneity	Total
Cisplatin	0.87	0.80–0.95	0.002	28%	HR = 0.85, 95%CI 0.78–0.92, P<0.0001, I^2^ = 27%
carboplatin	0.72	0.58–0.89	0.002	0%	
Asian people	0.80	0.69–0.94	0.005	50%	
non-Asian people	0.86	0.79–0.95	0.003	20%

### Progression-free survival

**Fig. 6:**
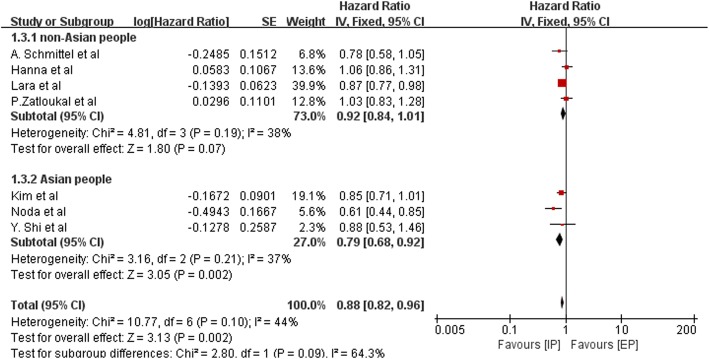
Forest plots estimating PFS stratified by ethnicity in IP vs EP

**Fig. 7:**
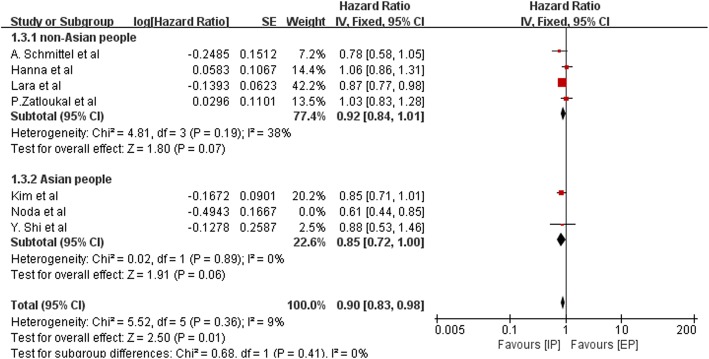
Sensitivity analysis of PFS was employed to explore sources of heterogeneity. Heterogeneity declined from I^2^ = 44% to I^2^ = 9% when the study performed by Noda et al. was excluded

HR for PFS was available for seven trials that included 2181 patients. The pooled HR for PFS was 0.88 (95% CI, 0.82–0.96; *P* = 0.002), and which was statistically significant. The fixed-effects model was adopted due to the mild heterogeneity (Chi^2^ = 10.77; *P* = 0.10; I^2^ = 44%). The results of subgroup analysis stratified by ethnicity (non-Asian or Asian patients) are shown in Fig. 6. We also performed sensitivity analysis and found that heterogeneity declined from I^2^ = 44% to I^2^ = 9% when the study performed by Noda et al. was excluded, however, the outcome was nearly unchanged (Fig. 7).

### Overall response, disease control, 1-year survival and 2-year survival rates

**Fig. 8:**
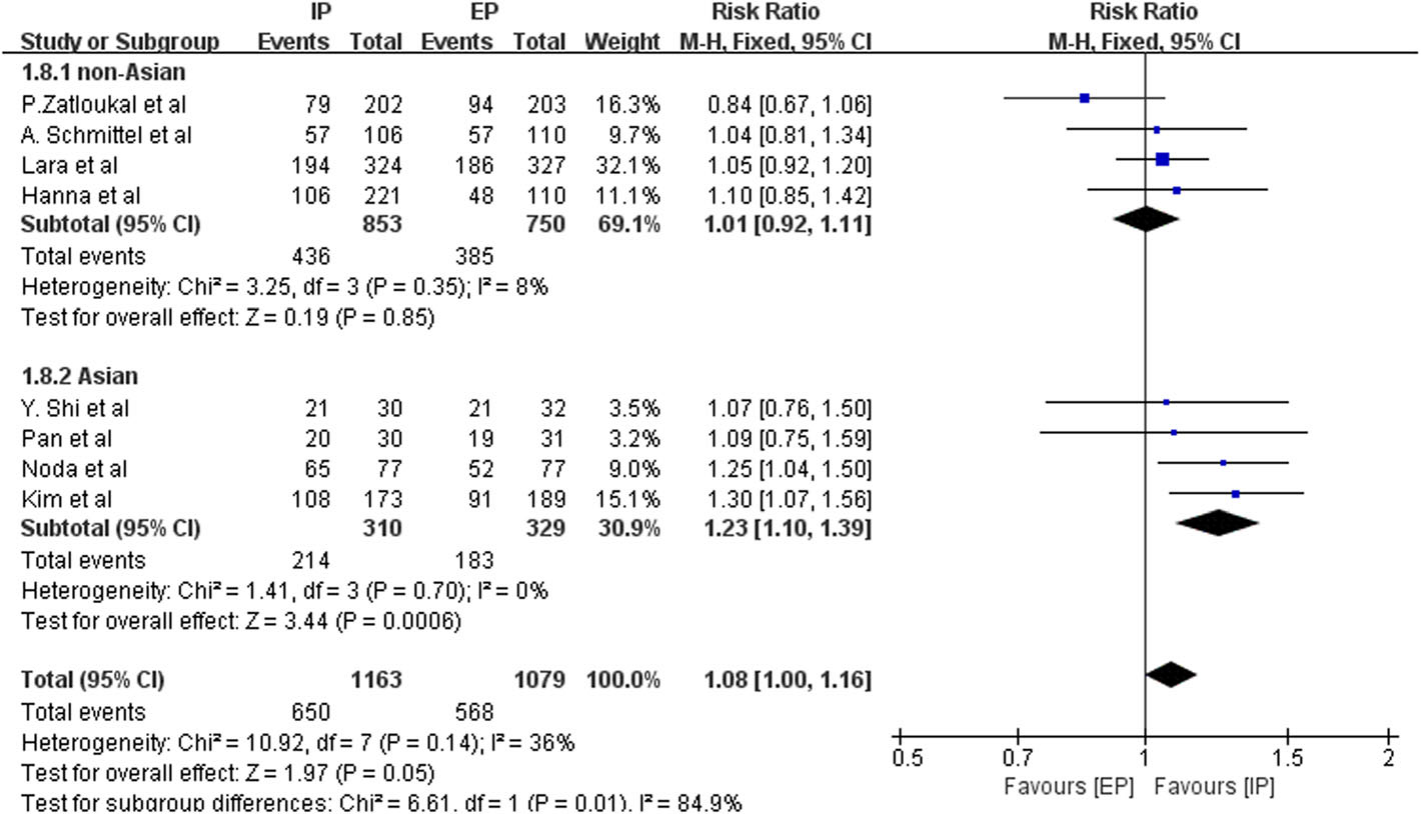
Forest plots estimating ORR stratified by ethnicity in IP vs EP

Data concerning overall response rate (ORR), disease control rate (DCR), 1-year survival rate, and 2-year survival rate were separately available for eight, seven, four, and three studies, respectively. The pooled RR of ORR was 1.08 (95% CI, 1.00–1.16; *P* = 0.05), which was statistically significant (Fig. 8). Heterogeneity was mild (Chi^2^ = 10.92; *P* = 0.14; I^2^ = 36%). The RR of the subgroup analysis with Asian patients was 1.23 (95% CI, 1.10–1.39) and was 1.01 with non-Asian patients (95% CI, 0.92–1.11). Significant discrepancies in RR of DCR and 1-year survival rate were not detected (Table 3). It is notable that the RR of the 2-year survival rate was 1.77 (95% CI 1.19–2.63; *P* = 0.01).

**Table 3:** The outcomes of RR, 95% CI, and I^2^ in CR, PR, ORR, DCR, 1-year survival rate, and 2-year survival rate

Analysis	Number Of Concerning Trials	Pooled RR	95%CI	P-Value	I^2^ For Homogeneity
CR	7	1.49	0.95–2.33	0.08	27%
PR	6	1.08	0.88–1.32	0.47	68%
ORR	8	1.08	1.00–1.16	0.05	36%
DCR	7	1.02	0.96–1.08	0.49	0%
1-Year Survival Rate	4	1.11	0.96–1.28	0.18	0%
2-Year Survival Rate	3	1.77	1.19–2.63	0.005	42%

DCR disease control rate

### Adverse effects

#### Hematological toxic effects

**Fig. 9:**
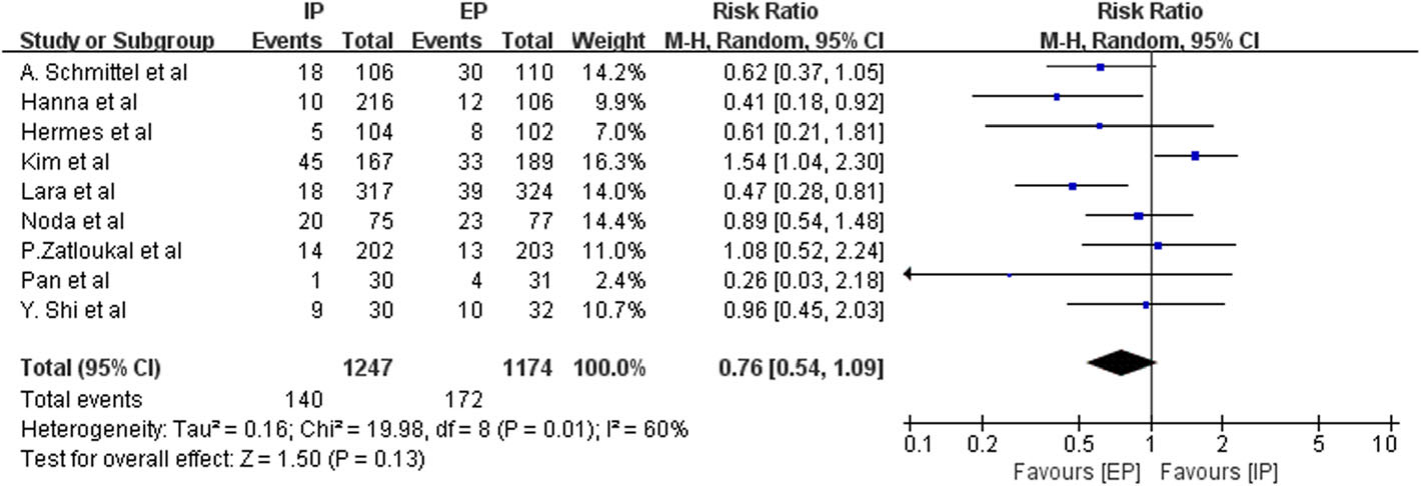
Forest plots estimating grade 3–4 anemia in IP vs EP

**Fig. 10:**
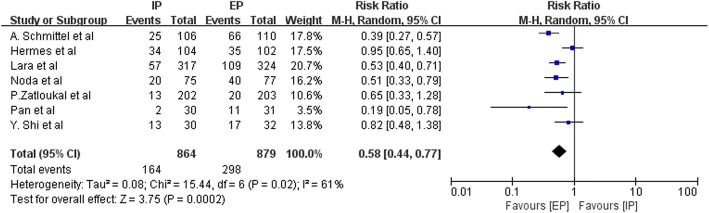
Forest plots estimating grade 3–4 leucopenia in IP vs EP

**Fig. 11:**
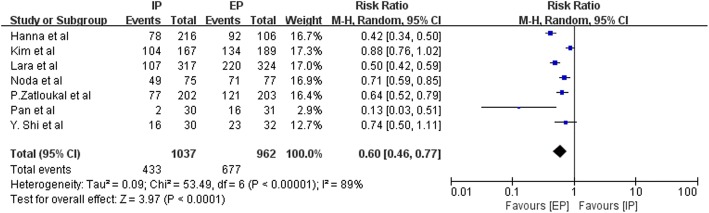
Forest plots estimating grade 3–4 neutropenia in IP vs EP

**Fig. 12:**
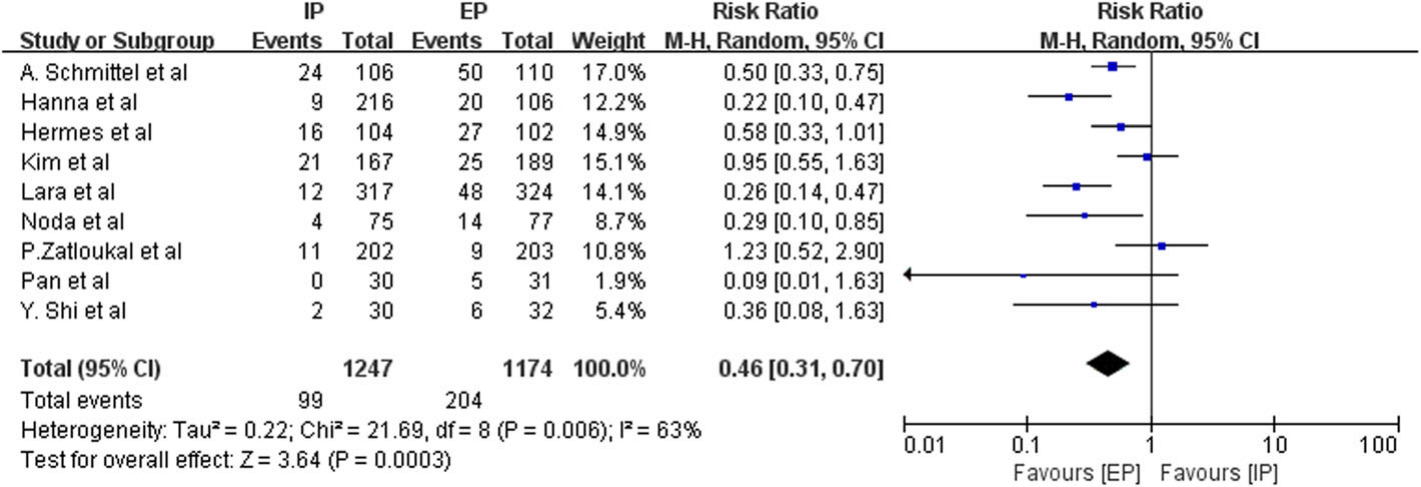
Forest plots estimating grade 3–4 thrombocytopenia in IP vs EP

**Fig. 13:**
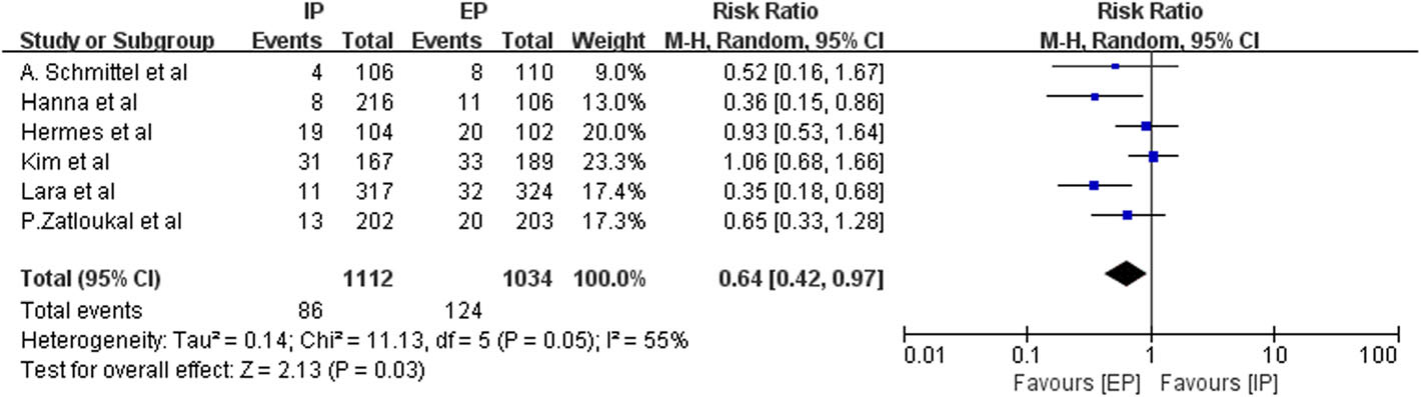
Forest plots estimating grade 3–4 febrile neutropenia in IP vs EP

Data on the frequency of National Cancer Institute Common Toxicity Criteria (NCI-CTC) grade 3–4 hematologic toxic effects, such as anemia, leucopenia, neutropenia, thrombocytopenia, and febrile neutropenia, were available from three to nine studies. Figures 9, 10, 11, 12 and 13 summarize the toxicity results. Patients treated with EP regimens were at a higher risk of grade 3–4 anemia (pooled RR = 0.76; 95% CI, 0.54–1.09; *P* = 0.13), grade 3–4 leucopenia (pooled RR = 0.58; 95% CI, 0.44–0.77; *P* = 0.0002), grade 3–4 neutropenia (pooled RR = 0.60; 95% CI, 0.46–0.77; *P* < 0.0001), grade 3–4 thrombocytopenia (pooled RR = 0.46; 95% CI, 0.31–0.70, *P* = 0.0003), and grade 3–4 febrile neutropenia (pooled RR = 0.64; 95% CI, 0.42–0.97; *P* = 0.03). Due to the heterogeneity regarding grade 3–4 anemia (Tau^2^ = 0.16; Chi^2^ = 19.98; *P* = 0.01; I^2^ = 60%), grade 3–4 leucopenia (Tau^2^ = 0.08; Chi^2^ = 15.44; *P* = 0.02; I^2^ = 61%), grade 3–4 neutropenia (Tau^2^ = 0.09; Chi^2^ = 53.49; *P* < 0.00001; I^2^ = 89%), grade 3–4 thrombocytopenia (Tau^2^ = 0.22; Chi^2^ = 21.69; *P* = 0.006; I^2^ = 63%), and grade 3–4 febrile neutropenia (Tau^2^ = 0.14; Chi^2^ = 11.13; *P* = 0.05; I^2^ = 55%) were obvious, the random-effects models were used.

#### Non-hematological toxic effects

**Fig. 14:**
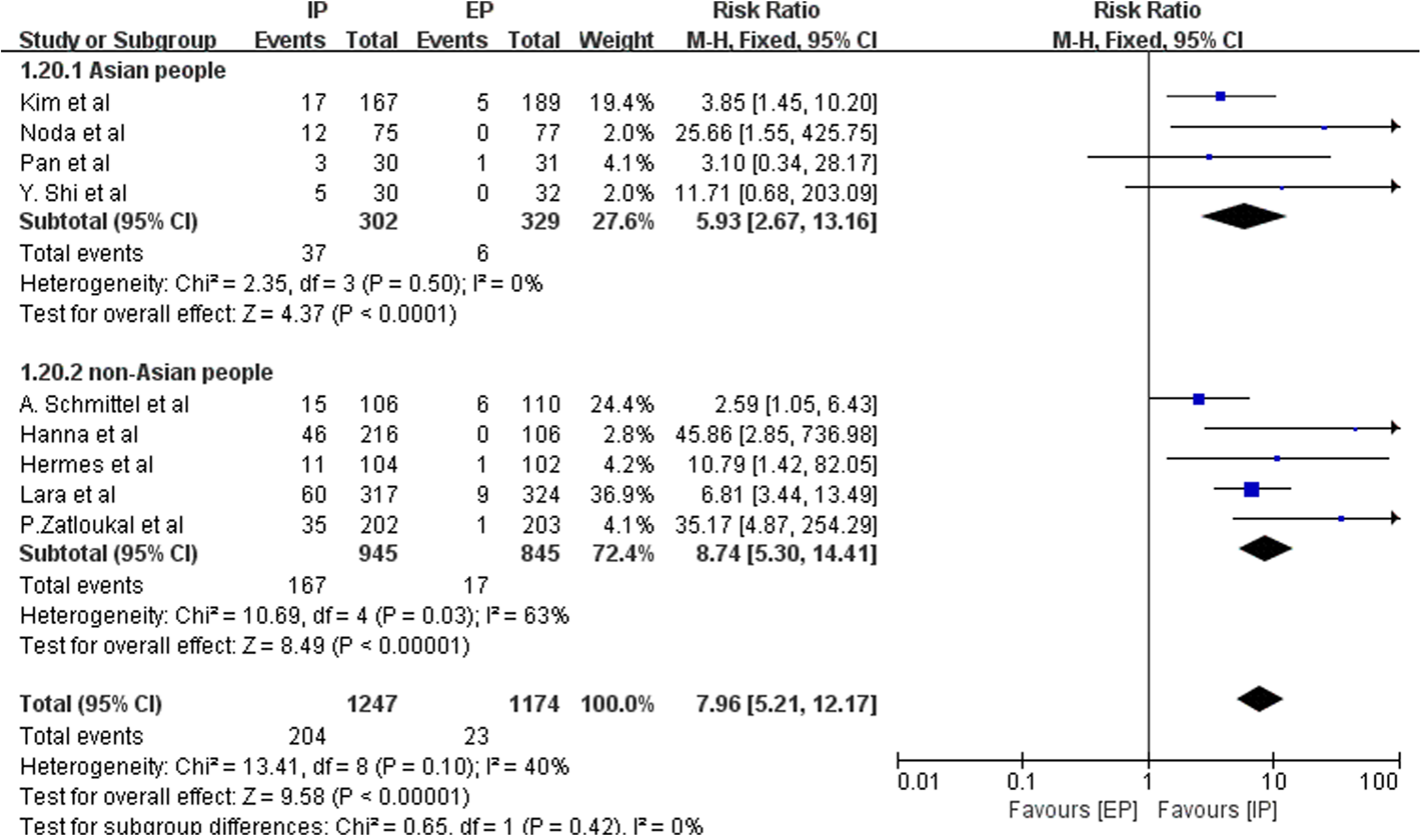
Forest plots estimating grade 3–4 diarrhea stratified by ethnicity in IP vs EP

**Fig. 15:**
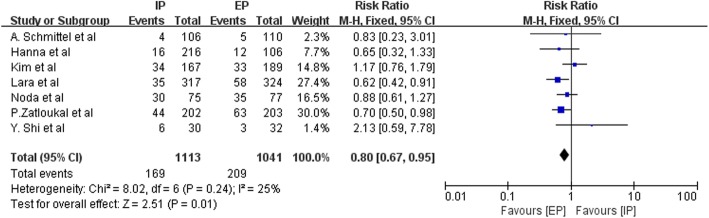
Forest plots estimating infection in IP vs EP

All trials reported grade 3–4 diarrhea, seven reported infection, four reported fatigue, and three reported alopecia and drug-related deaths. Figures 14 and 15 presented the results of grade 3–4 diarrhea and infection. An IP chemotherapy regimen led to more grade 3–4 diarrhea (pooled RR = 7.96 95% CI, 5.21–12.17; P < 0.00001) and less infection (pooled RR = 0.80; 95% CI, 0.67–0.95; P = 0.01). On the other hand, differences in the incidence of alopecia (pooled RR = 0.48; 95% CI, 0.18–1.29; *P* = 0.15), fatigue (pooled RR = 1.18; 95% CI, 0.98–1.42; *P* = 0.07), and drug-related death (pooled RR = 1.53; 95% CI, 0.79–2.99; *P* = 0.21) were not statistically significant between patients treated with an IP regimen as compared to those who were treated with an EP regimen. The details of all the toxic effects were illustrated in Table 4.

**Table 4:** Toxicity outcomes in this meta-analysis

Adverse Effects		Number Of Concerning Trials	Pooled RR	95%CI	P-Value	I^2^ For Homogeneity
Hematological Toxic Effects	Grade 3–4 Anemia	9	0.76	0.54–1.09	0.13	60%
	Grade 3–4 Leucopenia	7	0.58	0.44–0.77	0.0002	61%
	Grade 3–4 Neutropenia	7	0.60	0.46–0.77	< 0.0001	89%
	Grade 3–4 Thrombocytopenia	9	0.46	0.31–0.70	0.0003	63%
	Grade 3–4 Febrile Neutropenia	6	0.64	0.42–0.97	0.03	55%
Non-hematological Toxic Effects	Grade 3–4 Diarrhea	9	7.96	5.21–12.17	< 0.00001	40%
	Infection	7	0.80	0.67–0.95	0.01	25%
	Alopecia	3	0.48	0.18–1.29	0.15	88%
	Fatigue	4	1.18	0.98–1.42	0.07	0%
	Drug-related Death	3	1.53	0.79–2.99	0.21	0%

## Discussion

Chemotherapy is an essential component of appropriate treatment for patients with SCLC [18]. The current standard treatment is chemotherapy with or without local radiotherapy for patients with SCLC who have a good performance status (0–2), as recommended by the National comprehensive cancer network guidelines as category 1 evidence. EP is the most commonly used chemotherapy regimen. This regimen provides response rates of 60% to 80%, with a median survival time of 8 to 10 months. Thus, chemotherapeutic agents with greater activity are urgently needed.

JCOG previously reported the results of a randomized phase III trial (JCOG9511). They found that irinotecan, an inhibitor of the nuclear enzyme topoisomerase I, could improve OS and PFS when combined with platinum. Nevertheless, a series of studies conducted in America and Europe failed to confirm these positive results [9–12]. More rigorous studies were included in this meta-analysis to further compare efficacy and toxicity between IP and EP regimens; we subsequently analyzed the combined results thereof within the various subgroups.

In this meta-analysis, IP and EP regimens were compared in terms of OS, PFS, ORR, DCR, 1-year survival rate, 2-year survival rate, and common toxic adverse events. We found that an IP regimen significantly improves OS as compared to an EP regimen in ED-SCLC patients. When stratifying subgroup analysis by platinum type and ethnicity, OS results were consistent with the overall results. However, we found that the HRs were lower in patients treated with carboplatin and in Asian patients. These data indicate that irinotecan is superior to etoposide in combination with carboplatin-based chemotherapy, and that Asian patients receive a greater benefit from an IP regimen.

The OS of the patients who received follow-up treatment could be influenced and this may explain the inconspicuous superior result. PFS as a more meaningful measure of treatment effects, a superior outcome of IP treatment was found. That is to say, the IP regimen showed a increase in PFS, and the difference was statistically significant. When we performed subgroup analysis stratified by ethnicity, we found that the HR for Asian patients was 0.79, which was statistically significant (*P* = 0.002, 95% CI, 0.68–0.92). The HR for non-Asian patients was 0.92 (95% CI, 0.84–1.01), indicating that the IP and EP regimens led to comparable PFS in this subgroup. This is probably because a reduction of irinotecan often occurs in non-Asian patients who more frequently carry the UGT1A1*28 allele and are thus at an increased risk for severe diarrhea [19, 20]. Thus, the efficacy of irinotecan might be influenced by dose reduction in non-Asian patients.

Sensitivity analysis was performed excluding the Noda trial (JCOG9511), which prematurely concluded after interim analysis because they found significant differences in OS, and reduced heterogeneity (in OS: *P* = 0.51, I^2^ = 0%; in PFS: *P* = 0.36, I^2^ = 9%). The HRs, which were 0.87 for OS (95% CI, 0.80–0.94; *P* = 0.0008) and 0.90 for PFS (95% CI, 0.83–0.98; *P* = 0.01), were almost in line with the overall results. In addition, a different extent of dose reduction was present in each study. Therefore, we conclude that the trial conducted by Noda et al. (JCOG9511) and the various doses of chemotherapy regimens used in various countries might account for some of the observed heterogeneity in our meta-analysis.

That the pooled RR showed superior ORR of IP regimen implies that more patients will respond to chemotherapy when treated with an IP regimen, especially for Asian patients. Differences in DCR and 1-year survival rate were not statistically significant. Moreover, we found that irinotecan was superior to etoposide in 2-year survival rate. However, the outcome of relatively higher RR for 2-year survival rate warrants further discussion due to the low number of studies and recruited patients.

Toxicity analyses indicated that more patients treated with an IP regimen were likely to experience grade 3–4 diarrhea, and fewer experienced grade 3–4 hematologic toxic effects than those treated with an EP regimen. These results are in agreement with those of previous studies and the meta-analysis of safety of IP and EP [21]. We also performed subgroup analysis to explore diarrhea as an adverse event. The pooled RR in Asian patients was 5.93 (95% CI, 2.67–13.16; P < 0.0001) and 8.74 in non-Asian patients (95% CI, 5.30–14.41; *P* < 0.00001). This indicates that non-Asian patients are more likely to experience grade 3–4 diarrhea. However, the difference was not statistically significant (Chi^2^ = 0.65; df = 1; *P* = 0.42; I^2^ = 0%). This difference occurred might because the aforementioned UGT1A1*28 genotype, which bears a lower allele frequency in Asians than in Caucasians [19], confers a marked increase in irinotecan-induced grade 3–4 diarrhea [20]. Thus, a dose reduction of irinotecan is more likely to occur in Caucasians. Meanwhile some in vitro studies indicated that gene polymorphisms in the UGT1A1*6 gene were also associated with irinotecan metabolism [22, 23]. The frequency of the UGT1A1*6 mutant genotype was higher in Asian patients than in Caucasians [22]. A meta-analysis by Cheng et al. demonstrated that the heterozygous variant of UGT1A1*6 showed no significant risk for severe diarrhea, while there was a significant risk associated with the homozygous variant [24]. Therefore, we speculate that the UGT1A1*6 gene polymorphism may have an impact on the development of irinotecan-induced diarrhea in the Asian population. Confounding factors, such as differing doses of irinotecan, and the UGT1A1 gene polymorphism may be the reasons why there was no significant association between ethnicities and development of grade 3–4 diarrhea in populations.

We believe that the strength of this study lies in the fact that we conducted a quality assessment to guarantee that studies of a higher quality were included in the meta-analysis. Furthermore, we performed subgroup analyses of both ethnicity and platinum. Finally, the results were therefore more robust and reliable due to the consequence of sensitivity analysis.

A potential limitation of this meta-analysis is related to the different doses of chemotherapy regimens, and the performance status thereof in the included trials. A lack of information regarding the detailed dosage and performance status information for each of the groups meant that we could not perform the respective subgroup analyses. Another possible bias may have been introduced by the study conducted by Noda et al., which might lead to an overly optimistic result due to its premature conclusion. Additionally, more individual patient data were needed to conduct our meta-analysis, as extracting data from a survival curve inevitably introduced bias.

## Conclusions

In summary, for patients with ED-SCLC who have a poor prognosis, the question of which regimen to use is a relevant clinical issue requiring consideration of several factors. Given that IP regimens improved OS, PFS, and ORR as compared to EP regimens, particularly for Asian patients, we conclude that IP regimens can confer a survival benefit. Patients who were treated with an IP regimen experienced grade 3–4 diarrhea more frequently, including fatal diarrhea, and experienced fewer hematologic toxic events that were generally manageable and reversible with the application of corresponding symptomatic treatment drugs. For this reason, toxic events might be a vital factor in regimen selection. We conclude that IP regimens may substitute for EP regimens, particularly for ED-SCLC patients who have a good performance status.

## Abbreviations

CI: confidence interval
DCR: disease control rate
EP: Etoposide in combination with cisplatin
ES-SCLC: extensive-staged small cell lung cancer
HR: hazard ratio
IP: Irinotecan in combination with cisplastin
ORR: overall response rate
OS: overall survival
PFS: progression-free survival
RR: relative risk
SCLC: small cell lung cancer
